# Unbiased screen for pathogens in human paraffin-embedded tissue samples by whole genome sequencing and metagenomics

**DOI:** 10.3389/fcimb.2022.968135

**Published:** 2022-09-20

**Authors:** Ronny Nienhold, Nadine Mensah, Angela Frank, Anne Graber, Jacqueline Koike, Nathalie Schwab, Claudia Hernach, Veronika Zsikla, Niels Willi, Gieri Cathomas, Baptiste Hamelin, Susanne Graf, Tobias Junt, Kirsten D. Mertz

**Affiliations:** ^1^ Institute of Pathology, Cantonal Hospital Baselland, Liestal, Switzerland; ^2^ Central Laboratory, Cantonal Hospital Baselland, Liestal, Switzerland; ^3^ Autoimmunity, Transplantation and Inflammation, Novartis Institutes for BioMedical Research (NIBR), Basel, Switzerland

**Keywords:** pathology, infection, pathogen identification, metagenomics, next-generation sequencing

## Abstract

Identification of bacterial pathogens in formalin fixed, paraffin embedded (FFPE) tissue samples is limited to targeted and resource-intensive methods such as sequential PCR analyses. To enable unbiased screening for pathogens in FFPE tissue samples, we established a whole genome sequencing (WGS) method that combines shotgun sequencing and metagenomics for taxonomic identification of bacterial pathogens after subtraction of human genomic reads. To validate the assay, we analyzed more than 100 samples of known composition as well as FFPE lung autopsy tissues with and without histological signs of infections. Metagenomics analysis confirmed the pathogenic species that were previously identified by species-specific PCR in 62% of samples, showing that metagenomics is less sensitive than species-specific PCR. On the other hand, metagenomics analysis identified pathogens in samples, which had been tested negative for multiple common microorganisms and showed histological signs of infection. This highlights the ability of this assay to screen for unknown pathogens and detect multi-microbial infections which is not possible by histomorphology and species-specific PCR alone.

## Introduction

In pathology, infections are routinely diagnosed through histomorphologic examination of formalin-fixed, paraffin-embedded (FFPE) tissues. For example, acid-fast bacteria such as *Mycobacterium tuberculosis* can be detected by Ziehl-Neelson staining or by immunohistochemistry (Crothers et al., 2021). *Herpesviridae* such as Herpes simplex viruses 1 and 2, varicella zoster virus or *cytomegalovirus* can also be detected by immunohistochemistry and Epstein-Barr virus by *in situ* hybridization (Solomon et al., 2017). These methods require careful screening of the whole slide by a pathologist, because a single signal is already diagnostically relevant. Therefore, immunohistochemistry is frequently accompanied by molecular methods such as specific-specific PCR assays, which enable the detection of individual bacterial DNA molecules even when diluted in vast amounts of host DNA (Guiver et al., 2001; Guy et al., 2003; Kim et al., 2015; Salehi et al., 2016; Liu et al., 2017; Nunes et al., 2018). However, nucleic acid yields from FFPE tissues are often low due to formalin fixation (Dietrich et al., 2013; Do and Dobrovic, 2015). Formalin fixation of tissues samples in pathology is required for the preservation of tissue morphology, but results in DNA fragmentation and formalin-induced sequence artifacts.

Lack of commercial assays for identification of common pathogens from infected tissue samples led to the implementation of laboratory developed tests (LDT) (van Coppenraet et al., 2007; Klaus et al., 2010; Rabelo-Goncalves et al., 2014). This includes pathogen-specific PCRs (Ogredici et al., 2010; Kiss et al., 2016) or sequence analysis of genus-specific amplicons (Fehr et al., 2020). These tests require lengthy validation procedures and their sensitivity and specificity varies between different institutions.

Currently, pathogens on FFPE tissues are routinely identified by sequential rounds of species-specific LDTs. This is a resource-intensive procedure that is limited by the amount of DNA from small tissue biopsies. Furthermore, LDTs are only available for a finite number of pathogens, which increases the probability of rare infectious agents remaining unidentified. A single assay for broad and unbiased detection of pathogens from FFPE tissues would enable a faster and more informative diagnosis.

In recent years, 16S rRNA gene sequencing has become a widely used method for identification of various bacteria. The 16S rRNA gene contains nine variable regions, separated by conserved regions. Taxonomic resolution of bacterial strains depends on the size and the localization of the analyzed 16S rRNA gene region (Johnson et al., 2019; Jeong et al., 2021). While full-length analysis of the gene can be performed on microbial cultures or soil samples, FFPE tissue samples only allow for partial 16S rRNA analysis due to the high-level fragmentation of DNA extracted from FFPE tissue samples. Full-length and partial 16S rRNA analysis show similar results down to genus level (Johnson et al., 2019). In contrast to full-length 16S rRNA sequencing, which enables discrimination of nearly all known bacterial species, partial analysis of the 16S rRNA gene only allows to identify a subset of bacteria at species level. Furthermore, this subset is highly dependent on the length of the analyzed gene region. To overcome this limitation, commercially available kits for 16S rRNA analysis often contain various amplicons covering multiple variable regions (Johnson et al., 2019).

Our previous attempts at using commercial 16S rRNA kits yielded variable results on FFPE tissues with bacterial infections, and many commercial kits do not include lists of detectable bacterial species. This prompted us to develop a more robust assay for detection of a broad range of bacterial species from infected FFPE tissues of variable quality. Here we present the first metagenomics analysis for unbiased detection and identification of bacterial pathogens from FFPE tissue samples. This cost-effective assay uses standard reagents and third-party software for analysis. This approach relies on local data management and thus avoids the data security and privacy issue of online or remote server-based metagenomics services, which makes it suitable for routine diagnostic use.

## Material and methods

### Ethics statement

This study was conducted according to the principles expressed in the Declaration of Helsinki. Ethics approval was obtained from the Ethics Committee of Northwestern and Central Switzerland (Project-ID 2020-00629). For all patients, either personal and/or family consent was obtained for autopsies and sample collection.

### Patients and sample collection

The study is based on the analysis of lung autopsy tissues collected at the Institute of Pathology Liestal, Switzerland. The first set of 30 samples from 25 individual patients was collected during the first wave of the COVID-19 pandemic between March and May 2020. The second set of 43 samples from 37 individual patients was collected during the second wave of the COVID-19 pandemic between October 2020 and January 2021. Clinicopathological features of all autopsy patients including symptoms, course of disease and comorbidities were described previously (Schwab et al., 2022).

### Nucleic acid extraction

Extraction of DNA from up to 10 sections of FFPE tissue samples was automated by EZ1 Advanced XL (Qiagen, Hilden, Germany) using the EZ1 DNA Tissue Kit (Qiagen, Hilden, Germany). Concentration of DNA was measured with Qubit 2.0 Fluorometer and Qubit dsDNA HS Assay (Thermo Fisher Scientific, Waltham, MA, USA).

### Round robin test samples

Round robin test 1 (samples 1-4) is the 2020 version of the BakNAT1 (RfB, Bonn, Germany) provided to test for genomic detection of *Bordetella pertussis*, *Chlamydia pneumoniae*, *Chlamydia trachomatis*, *Mycoplasma pneumoniae*, *Coxiella burnetii*, *Francisella tularensis*, *Legionella pneumophila* and *Staphylococcus aureus*. Round robin tests 2 and 3 are tests for the specific detection of *Helicobacter pylori* (samples 5-8) and *Borrelia burgdorferi sensu lato* (samples 9-12), respectively (INSTANT e.V., Düsseldorf, Germany). Round robin test 4 is the PolyVir21/21 test for the specific detection of *adenoviruses*, *herpes viruses* and *papillomaviruses* (RfB, Bonn, Germany). All round robin test samples were reconstituted according to the instructions of the provider, and DNA was extracted as described above. The results of all round robin tests were confirmed by the providers (RfB, Bonn, Germany; INSTANT e.V., Düsseldorf, Germany) with written certificates that can be provided upon request.

### Generation and dilution of mock communities

Three bacterial mock communities were generated from 18 individual clinical isolates from patients presenting with a respective infection. Individual bacterial species were isolated and characterized by matrix-assisted laser desorption/ionisation time-of-flight mass spectrometry (MALDI-TOF MS, **Supplementary Table 1**). DNA from these 18 bacteria was isolated and randomized to three mock communities of 6 species each at 100’000 genomes per species. Six serial 1:10 dilutions of mock communities were generated. Purified human genomic DNA extracted from normal FFPE lung tissues from two autopsy patients was mixed at a 1:1 ratio and spiked into the serial dilutions of bacterial mock communities at 100’000 genomes each. In addition, a forth mock community was generated using a microbial community standard containing 8 different bacterial species (Zymo Research, Tustin, CA, USA) which was diluted as described before.

### Detection of pathogens by metagenomics whole genome sequencing

To identify potential pathogens by metagenomics whole genome sequencing, 250ng of genomic DNA was enzymatically cleaved and barcoded using the Ion Xpress Plus Fragment Library Kit (Thermo Fisher Scientific, Waltham, MA, USA). Subsequently, the libraries were quantified (Ion Library TaqMan Quantitation Kit, Thermo Fisher Scientific, Waltham, MA, USA) and analyzed with Ion GeneStudio S5xl (Thermo Fisher Scientific, Waltham, MA, USA). Sequencing data for each sample was analyzed using the CLC genomics workbench (version 21.0.2, Qiagen, Hilden, Germany) in combination with the microbial genomics module (version 21.1, Qiagen, Hilden, Germany): The raw reads were trimmed by quality (Mott algorithm with limit 0.05 and a maximum of 2 ambiguous bases per read) and mapped to the human genome (GRCh37 hg19, match score: 1, mismatch cost: 2, indel opening cost: 6, indel extension cost: 1). Recent advantages in telomere-to-telomere sequencing, however, resulted in a new, improved and, as authors claim, gapless assembly of the human genome (Nurk et al., 2022). Therefore, it is possible that a number of reads, which we call “unidentified”, originate from genomic regions that were poorly annotated in the human genome (GRCh37 hg19). Unmapped reads were analyzed by taxonomic profiling to identify reads of bacterial or viral origin. Profiling relied on an index of 5’868 bacterial reference genomes with a minimum length of 100’000 bp and 11’535 viral genomes with a minimum length of 1’000 bp, retrieved from the NCBI Reference Sequence Database (date of download: 2021-01-21). These length cut-offs were introduced to avoid matches to incomplete database entries for bacterial and viral genomes. The NCBI Reference Sequence Database also includes DNA reference information of fungal and archae pathogens, but lacks reference genomes of the protozoan kingdom. As the focus of this study was detection of bacteria and DNA viruses, we did not optimize the method for fungi yet.

### Down-sampling *in silico*

For metagenomics whole genome sequencing, a median of 25 million total reads per sample was generated and analyzed. To make the method more cost-effective for routine use without loss of quality, we tested *in silico* whether 5 million reads are sufficient for the analysis. To that end, 5 million reads were randomly selected from the raw reads of 6 FFPE samples (**Supplementary Figure 1**) and subjected to our metagenomics analysis pipeline. This process was repeated 100 times, and the results were compared to the results from the full set of reads. This down-sampling to 5 million reads was performed in order to reach costs comparable to a small or medium NGS sequencing panel as is frequently offered in most molecular pathology labs.

### Detection of individual respiratory pathogens by quantitative PCR

RNA was extracted from up to six sections of FFPE lung tissue blocks using RecoverAll Total Nucleic Acid Isolation Kit (Thermo Fisher Scientific, Waltham, MA, USA). Syndromic testing for respiratory pathogens was performed using the TaqMan Microbial Array Specialty Card, which represents an early access version of the TrueMark Respiratory Panel 2.0 (ThermoFisher Scientific, Waltham, MA, USA). This 384 well plate allows the analysis of eight samples in parallel for 42 respiratory pathogens by TaqMan (**Supplementary Table 2**). For each sample, 80 ng of total RNA were converted to cDNA, pre-amplified, applied to the TaqMan array card and measured by the QuantStudio 7 Pro Real-Time PCR System (ThermoFisher Scientific, Waltham, MA, USA).

### Assessment of lung damage and neutrophilic infiltration in FFPE tissue samples

Hematoxylin and eosin (H&E) and Elastica van Gieson (EvG) stained sections of all lung tissues used in this study were independently evaluated by two experienced board-certified pathologists (VZ and KDM) for the presence of diffuse alveolar damage (DAD) and its stage, intra-alveolar edema and hemorrhage. In addition, the general severity of histopathological changes was scored for all 73 lung samples (1=mild/discrete alterations, 2=moderate, 3=severe changes). Parameters that were taken into account for scoring included reduction of alveolar air-filled spaces, typical histologic features of DAD with hyaline membrane formation, infiltration of lymphocytes, monocytes and neutrophils into interstitial and alveolar spaces, type 2 pneumocyte hyperplasia, desquamation of pneumocytes, histologic features of organizing pneumonia including intra-alveolar fibrin deposition and fibrosis (acute fibrinous and organizing pneumonia, AFOP) (Copin et al., 2020; Xu et al., 2020).

The number of neutrophils per lung tissue section was scored by two experienced board-certified pathologists on H&E stained sections and by immunohistochemical stains for CD15 and MPO (0=no neutrophils, 1=few neutrophils, 2=moderate number of neutrophils, 3=high number of neutrophils). Discrepant cases were reviewed by a third board-certified pathologist to reach consent.

### Statistical analysis

All statistical analyses were performed within GraphPad Prism v9.2. *, p < 0.05; **, p < 0.01; ***, p < 0.001. Groups were compared using one-way analysis of variance (ANOVA) or Chi-square test.

## Results

**Figure 1:**
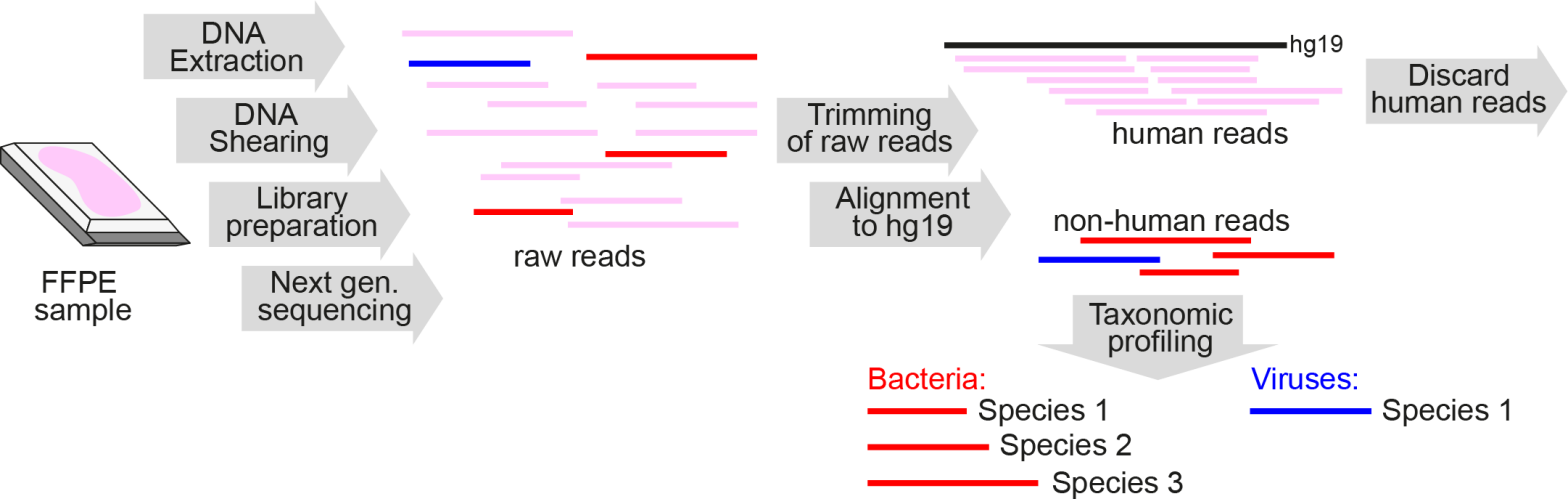
Schematic overview of metagenomics analysis of DNA extracted from FFPE tissue samples. FFPE, formalin-fixed paraffin-embedded; Next gen. sequencing, Next-generation sequencing.

We developed a novel workflow for identification of pathogens from formalin-fixed, paraffin-embedded (FFPE) tissue samples (**Figure 1**) and validated our protocol by analyzing four DNA samples containing bacterial mock communities, three round robin test sample sets and 73 FFPE lung autopsy tissues from 62 patients who died during the first and second wave of the COVID-19 pandemic and underwent autopsy at our institution (Schwab et al., 2022).

### Metagenomics workflow on test samples of known composition

**Figure 2:**
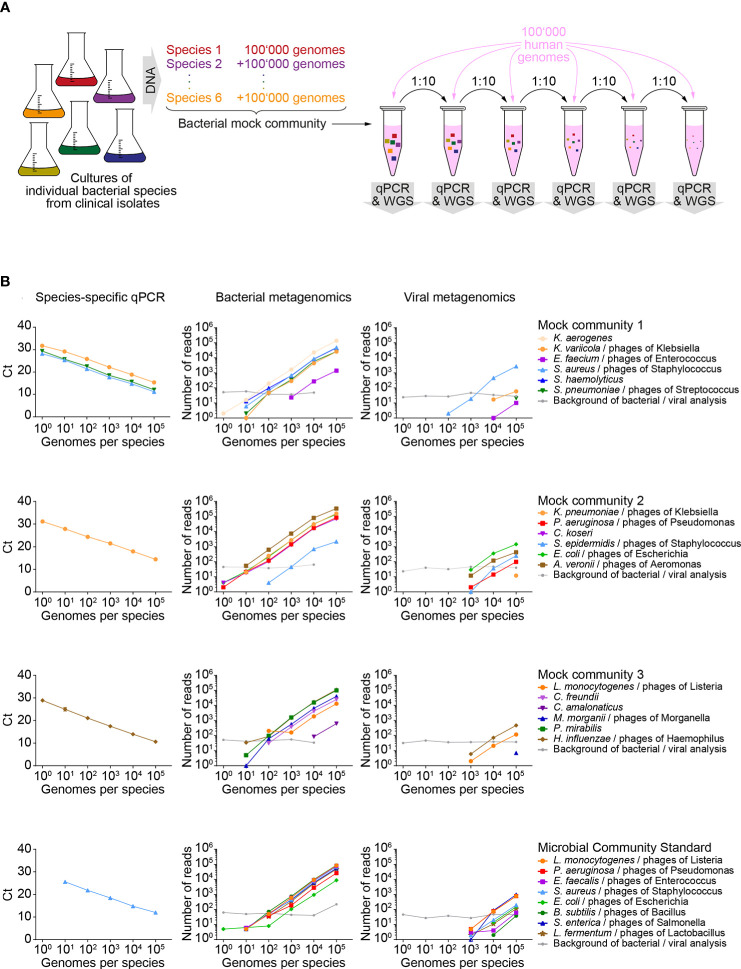
Sensitivity of metagenomics analysis on test samples of known composition. **(A)** Schematic workflow for generating serial dilutions of known bacterial pathogens (mock communities), admixed with human genomic DNA. **(B)** Detection of known bacterial pathogens and associated phages in diluted mock communities and detection of reference strains in a microbial community standard by quantitative PCR (qPCR, left column of panels), taxonomic profiling for bacteria (middle column of panels) and viruses (right column of panels). Each row of panels represents results of an individual mock community represented by several dilutions. Within a row of panels, line color and symbol shape indicate data originating from a specific isolate or reference species. Background of bacterial/viral analysis: The most abundant background signal detected for a bacterial and a viral species is indicated. WGS, whole genome sequencing; Ct, cycle threshold.

To establish our workflow, we used four DNA test samples of known composition, each containing serial dilutions of DNA from 6-8 known bacterial pathogens (so called mock communities) admixed with human genomic DNA (**Figure 2A**). These samples simulated DNA from patient lungs suffering from polymicrobial infections. Each of these four test samples contained at least one bacterial species detectable by a commercial multiplexed qPCR array for pathogen detection (**Supplementary Table 2**). Dose linearity of this assay and sensitivity down to 1 genome in 100’000 human genomes was observed for 5 out of the 6 covered species (**Figure 2B**, left column). The remaining species, *Staphylococcus aureus*, was detected down to 10 genomes in 100’000 human genomes. It is of note that this is not a direct indicator of sensitivity of the workflow on FFPE tissues, as the bacterial DNA was not isolated from FFPE bacteria.

The same DNA dilution series was analyzed by metagenomics. An average of 25x10^6^ (range 20x10^6^ - 29x10^6^) reads were generated per sample. A median of 99.9% of reads passed the quality based trimming and filtering (range 99.9 - 100.0%). Mapping to the human genome GRCh37 (hg19) excluded 99.6% of reads (range 92.2 - 99.7%) before entering taxonomic analyses (**Supplementary Figures 2A, B**). As expected, the highest levels of non-human reads were measured in test samples containing 100,000 bacterial genomes per species (median 3.7%, range 2.4% - 7.8%), while samples with lower content of bacterial DNA had a median of 0.4% (range 0.3-1.5%) of unmapped reads (**Supplementary Figure 2C**). Depending on the number of bacterial genomes per sample, 0.2% to 87.3% and 0.03% to 1.11% of the unmapped reads were taxonomically assigned to the bacterial or viral kingdom, respectively. Within each kingdom, the majority of reads could be assigned to bacterial (50.9 – 89.7%) or viral (59.3 – 100.0%) species. Of note, the viruses detected by our assay were all caudovirales, which represent DNA phages that are associated with at least one of the known bacterial species in the mock communities. Consistent with the composition of our test samples, we did not detect viruses that are pathogenic for humans. However, our data show that our metagenomics workflow is suitable for identification of viruses with a DNA genome.

Six bacterial species and three viruses were identified in over 50% of mock community dilution samples (**Supplementary Table 3**) and in the plain human genomic DNA sample, while they were absent from the undiluted bacterial mock communities. Therefore, we conclude that DNA from these species was co-purified with the human DNA samples that were used for dilution of bacterial mock community DNA. This endogenous background of bacteria and viruses served as an internal standard for our assay: We defined the number of reads from the most abundant background species for each of the two kingdoms, bacteria and viruses, as the minimum number of reads/threshold for species to be identified as pathogens.

Metagenomics analysis of mock communities resulted in dose-linear detection of most bacterial genomes (**Figure 2B**, middle column). In samples with 1 and 10 genomes per DNA species, 5 (19.2%) and 18 (69.2%) of all 27 bacterial genomes in our test samples were detected, but the signal was weaker than the microbial background introduced as a standard *via* the human DNA. In samples with 100 and 1’000 genomes per isolate, 24 (92.3%) and 25 (96.2%) of all bacterial species were detected, respectively. Hence, this data indicates an approximately 100-fold lower sensitivity of the metagenomics analysis compared to species-specific qPCRs (**Supplementary Figure 2D**). Taxonomic profiling revealed presence of DNA bacteriophages in samples with 1’000 or more bacterial genomes per isolate (**Figure 2B**, right column). In each mock community, detected species of phages matched with the bacterial genus of the included isolates.

### Metagenomics workflow on round robin test samples of unknown composition

**Figure 3:**
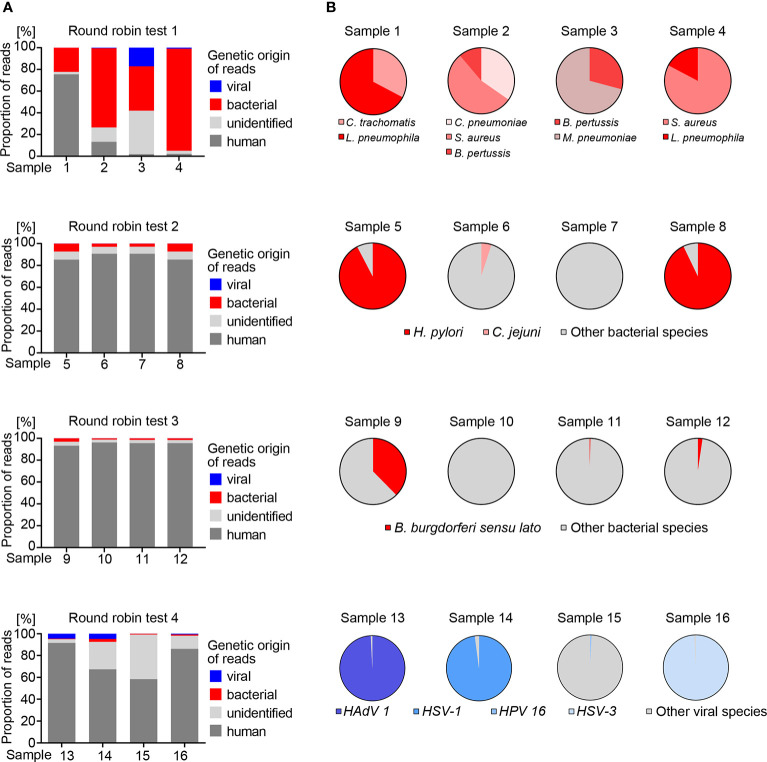
Bacterial and viral reference species identified by metagenomics analysis in round robin tests. **(A)** Genetic origin of sequence information interpreted from read mapping to the human genome GRCh37 (hg19) or taxonomic profiling of non-human reads. Reads that could not be assigned to human, bacteria or viruses were labeled as unidentified. **(B)** Different species identified in bacterial and viral reads in individual round robin test samples.

In order to test the utility of the metagenomics assay for DNA samples of unknown composition, the workflow was applied to three round robin tests containing multiple bacterial species, with each test consisting of four individual samples (**Figure 3**). Round robin test 1 was designed for detection of *Bordetella pertussis*, *Chlamydia pneumoniae*, *Chlamydia trachomatis*, *Mycoplasma pneumoniae*, *Coxiella burnetii*, *Francisella tularensis*, *Legionella pneumophila* and *Staphylococcus aureus*. Metagenomic analysis of the four samples revealed varying DNA amount (<10% to >70%) of human origin (**Figure 3A**). Taxonomic analysis of non-human reads revealed that each sample contained at least two species of interest, while *Coxiella burnetti* and *Francisella tularensis* were absent in all four samples (**Supplementary Table 4**). The result was confirmed by the provider of the round robin test, demonstrating that our metagenomics assay is suitable for parallel detection of unknown bacterial species. Of note, we detected *Torque teno sus virus* species 1a, 1b and k2b, i.e. pig anelloviruses with a single-stranded circular DNA genome, in 17.1% of the total reads of sample 3. This was again confirmed by the provider of the round robin test. Therefore, the high proportion of unidentified reads in this sample is likely attributable to swine tissue. Together with the detection of bacterial phages in mock communities, this allows us to draw the conclusion that our metagenomics assay is suitable for the detection of DNA viruses, but due to the nature of our pipeline that focuses on DNA analysis, we cannot detect RNA viruses.

Round robin test 2 was designed for detection of *Helicobacter pylori*. Metagenomics analysis detected *H. pylori* in two out of four samples, which were confirmed as positive by the provider. In addition, metagenomics identified *Campylobacter jejuni* in one *H. pylori* negative sample, and the provider confirmed its presence as a control for assay specificity. All samples of this round robin test contained 85.3% – 90.7% human DNA, and 2.8% – 7.3% and 0.005 – 0.018% bacterial and viral reads, respectively (**Figure 3A**).

The third round robin test was designed for detection of *Borrelia burgdorferi sensu lato*. Our metagenomics analysis assigned reads to species of the *Borrelia burgdorferi sensu lato* complex in three of the four samples at varying read numbers reflecting different concentrations of the analytes (**Supplementary Table 4**) (Rudenko et al., 2011). This was confirmed by the provider. A large fraction (85.3% - 96.2%) of human reads was found in all samples, and 1.2% – 3.1% and 0.009% – 0.017% bacterial and viral reads, respectively (**Figures 3A, B**). All samples of round robin tests 2 and 3 shared a similar pattern of 12 bacterial and 17 viral species, which was confirmed as endogenous background by the provider of these two round robin tests (**Supplementary Table 5**).

### Metagenomics workflow on human FFPE lung tissues

Finally, we used the metagenomics workflow to analyze 73 formalin-fixed, paraffin-embedded (FFPE) lung tissues from a cohort of 62 autopsy patients who all died during the first and second wave of the COVID-19 pandemic and underwent autopsy in our institution (Schwab et al., 2022). Twenty-three (37%) of these 62 patients died from COVID-19, mostly without bacterial co-infections, 11 (18%) SARS-CoV-2 negative patients were suffering from bacterial respiratory infections, and 4 (6%) of these 11 patients died from their bacterial lung infections. Seven (11%) of these 11 patients and the remaining 28 (45%) patients died of non-respiratory causes (n=35, 56% of patients). These autopsies were analyzed as two consecutive series of 30 lung tissue samples from 25 patients (set 1) and 43 lung tissue samples from 37 patients (set 2).

**Figure 4:**
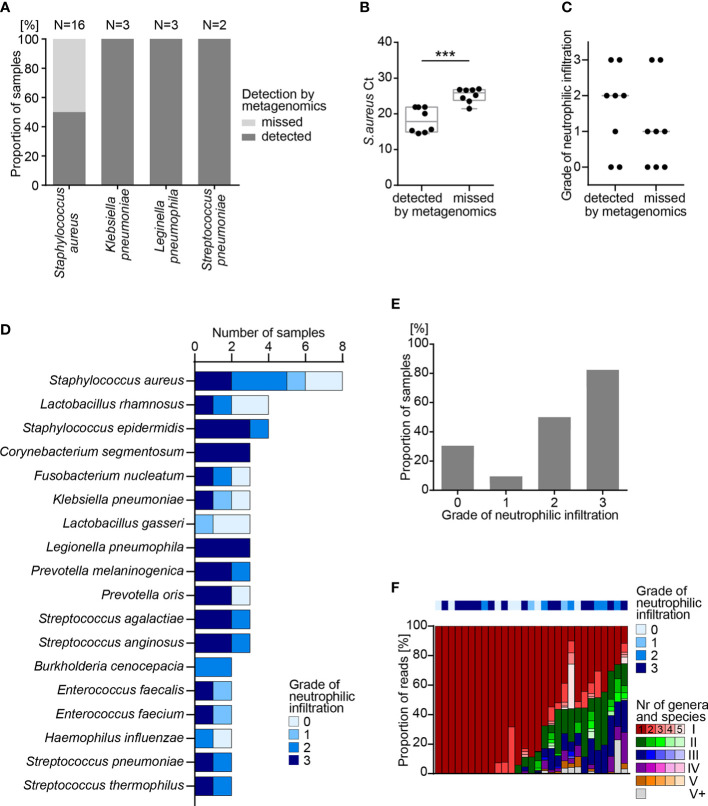
Metagenomics analysis of FFPE lung tissue samples. **(A)** Detection of bacterial pathogens in human FFPE lung tissue samples by quantitative PCR (qPCR) and by metagenomics analysis. **(B)** Detection of *S. aureus* by qPCR and by metagenomics analysis in 16 samples. Box shows range from first to third quartile and median, whiskers display minimum and maximum. Statistical analysis: T-test; ***, p<0.001. **(C)** Grade of neutrophilic infiltration in human FFPE lung autopsy tissues. **(D)** Number of samples positive for the indicated species, as determined by metagenomics analysis. **(E)** Fraction of samples with one or more bacterial species identified by metagenomics analysis, sorted by grade of neutrophilic infiltration. Statistical analysis: χ-square-test; ****, p<0.0001. **(F)** Fraction of reads representing individual species for each sample presented in E. Colors (e.g. red, green, blue, purple, orange) represent individual genera and shades of the same color (e.g. dark red, red, light red, rose, faint rose) represent individual species of the same genus, sorted from most frequent to least frequent. According to their rank of abundance within an individual sample, genus and species are highlighted by colors and shades: red represents the most frequent genus; green the 2^nd^, blue the 3^rd^, purple the 4^th^ and orange the 5^th^ most frequent genus. Grey represents all genera with rank 6 or higher. FFPE, formalin-fixed paraffin-embedded; Ct, cycle threshold.

All human lung tissue samples were screened for respiratory pathogens by the commercial multiplexed qPCR array card that was used as a control for the metagenomics assay on test samples of known composition. This assay revealed bacteria in 21 (29%) lung tissue samples of 19 (31%) cases. *Staphylococcus aureus* was most frequently detected (n=16 samples), while *Klebsiella pneumoniae*, *Legionella pneumophila* and *Streptococcus pneumoniae* were detected in only 3 and 2 samples, respectively (**Figure 4A**).

Next, all human lung tissue samples were screened for pathogens by the metagenomics assay. For the first set of samples (n=30), a median of 25x10^6^ total reads per sample was generated to allow comparison with results from mock communities. In order to reduce costs for future implementation in diagnostic routine, the second set of samples (n=43) was analyzed at a median of only 5x10^6^ total reads per sample. Down-sampling was performed and 100 times repeated for all samples positive for bacterial pathogens to show that reduction from 25x10^6^ to 5x10^6^ total reads has no significant impact on detection probability of pathogens in clinical samples (**Supplementary Figure 1**). Independent of the number of total reads per sample, the results for lung FFPE samples were comparable (**Supplementary Figures 3A, B**). As expected, lung FFPE tissue samples, on average, showed a larger proportion of human reads (99.4%) and a smaller fraction of bacterial (0.016%) or viral reads (0.0004%) than round robin test samples (**Supplementary Figure 3C**, **Supplementary Table 6**).

Metagenomics analysis identified pathogens in 13 (62%) of 21 lung tissue samples that showed a positive reading by the multiplexed qPCR array card (**Figure 4A**), yet failed to detect *S. aureus* in 8 (33.3%) samples. Ct values of *S. aureus*-specific qPCR indicated lower abundance of *S. aureus*, as indicated by a high Ct value in samples that escaped the metagenomics readout (**Figure 4B**). This dichotomy was consistent with a trend in the histological picture, in which neutrophilic infiltrates are indicative of bacterial inflammation. In 5 out of 8 (62.5%) samples with *S. aureus* detected by metagenomics, histology revealed moderate to high numbers of neutrophilic granulocytes, while only 2 out of 8 (25.0%) samples where metagenomics missed *S. aureus* showed signs of infiltrating neutrophils (**Figure 4C**).

As mentioned before, the commercial qPCR respiratory array card allows for detection of only 42 defined respiratory pathogens by TaqMan (**Supplementary Table 2**). In contrast, our metagenomics assay can detect more than 5’000 bacteria and more than 11’000 DNA viruses. While the qPCR respiratory array panel identified a limited number of 4 bacterial species in human FFPE lung samples, metagenomics detected more different pathogens. Most frequently, metagenomics analysis of lung FFPE tissues identified *S. aureus* (n=8), *Lactobacillus rhamnosus* (n=4) and *Staphylococcus epidermidis* (n=4) (**Figure 4D**). Some bacteria were known respiratory pathogens such as *S. aureus*, *K. pneumoniae*, *L. pneumophila* and *S. pneumoniae*, yet we also detected species of *Lactobacillus* and *Prevotella* (**Figure 4D**). These results indicate the potential of the metagenomics analysis to identify frequent as well as rare pathogens or commensals, which may have contaminated the lung tissues of deceased patients, e. g. as a consequence of aspiration and subsequent pneumonia.

To correlate histological signs of bacterial inflammation to metagenomics results, neutrophilic infiltration was scored for all lung autopsy tissues (**Supplementary Figure 3D**). Metagenomics analysis identified bacterial species in 29 (39.7%) of 73 lung tissue samples, and most of the samples with bacteria showed moderate to heavy neutrophilic infiltration of grades 2 and 3 (20 out of 29 cases, 69.0%, **Figure 4E**). In lung tissue samples with bacteria identified by metagenomics analysis, more than half of the bacterial reads could be assigned to a single bacterial species (19/29, 65.5%). In only 7 (24.1%) of 29 samples, taxonomic profiling assigned reads to 6 or more different genera (**Figure 4F**). This shows that the metagenomics workflow may serve to identify the bacterial diversity in an infected organ, e.g. during superinfections.

In summary, we established and validated a novel metagenomics assay as an approach for unbiased identification of unknown pathogens in routine diagnostic FFPE tissue samples. This approach is able to detect down to 1‰ of bacterial reads in a background of human genomes, i.e. 100 bacterial genomes in 100’000 human genomes. It complements histological readouts of infected samples. As such, it adds depth to pure morphological analysis and qPCR analysis. This assay may serve to appreciate (1) unidentified bacterial and viral (DNA) infections in FFPE tissues; (2) species of bacteria and DNA viruses in polymicrobial infections; (3) the tissue microbiome on FFPE samples.

## Discussion

Here we describe a novel metagenomics WGS method for unbiased identification of microbes in human FFPE tissue samples. We validated our workflow using DNA test samples of known composition (“mock communities”) and round robin test sample sets of unknown composition before analyzing human FFPE lung tissues from two different sets of unselected autopsies. A multiplexed panel of species-specific qPCRs covering 42 frequent respiratory pathogens was used as an orthogonal assay and demonstrated that metagenomics WGS identified bacterial species in samples of both known and unknown composition reliably. Comparison of the qPCR and metagenomics workflow further demonstrated that qPCR is, on average, a factor of 100 more sensitive than metagenomics, while metagenomics serves to appreciate the diversity of pathogens in FFPE tissues.

One of our most notable results was the identification of diverse bacterial species in human tissue samples with neutrophilic infiltration. This underscores the value of a diagnostic approach that integrates morphology and molecular analyses in daily practice. The vast majority of infected lung samples presents with a low diversity of infection. Most frequently, mono-microbial infections are observed, and polymicrobial infections are often dominated by one individual pathogen. Only in one FFPE lung tissue sample of an autopsy case, metagenomics analysis revealed a polymicrobial infection with a mixed composition of diverse pathogens, while the histological picture was similar to tissue samples infected with a single pathogen (**Supplementary Figure 4**). Despite formaldehyde-induced artifacts and low yields of FFPE DNA obtained from human lung tissue samples (Do and Dobrovic, 2015; Arreaza et al., 2016; McDonough et al., 2019), accuracy of detection was comparable to data from dilution series of mock communities and round robin tests.

As a second important insight, we found a 100x lower sensitivity of the metagenomics analysis compared to species-specific PCRs in FFPE lung tissues and mock communities. This lower sensitivity can be explained by the absence of pathogen-specific enrichment steps in the metagenomics workflow. For metagenomics, we intentionally avoided species-specific enrichment, e. g. by targeted amplification, to avoid selection bias for some species and reduced sensitivity for less abundant ones. As a means to increase overall sensitivity, we attempted enrichment of bacterial DNA by depletion of human DNA using methylated CpG-specific binding protein, a method that was described for body fluids with high bacterial load (Feehery et al., 2013). However, when tested on DNA from FFPE lung tissue samples, this enrichment method resulted in DNA yields that were insufficient for further processing, likely due to the low bacterial load of tissue (*data not shown*). As an alternative approach to increase sensitivity, pre-analytical microscopic examination of the specimen and selection of the area of interest could be useful in some cases. For example, based on our result that tissues with neutrophilic infiltration contained most bacteria, selection of neutrophil-rich tissue areas may increase the sensitivity of pathogen detection in FFPE samples (Selva et al., 2004). However, in light of the sensitivity limits of our assay, negative results of metagenomics analyses in samples with a high grade of neutrophilic infiltration do not exclude the presence of bacterial species, especially low-titer infections or pathogens difficult to diagnose (e.g. with *Mycobacterium tuberculosis*), or the presence of other pathogens such as fungi or protozoa, which are not covered by the reference database (Yeager et al., 1967; Mustafa et al., 2006; Lee et al., 2011).

The lower sensitivity of the metagenomics method, however, has one advantage over sensitive species-specific qPCR assays. Sub-clinical infections or commensals with low abundance are not detected. With that, the metagenomics assay reduces false-positive signals and the associated risk of over-interpretation of findings. Especially in an autopsy setting, it is key to distinguish post-mortal bacterial growth from an active infection before death.

Detection of bacteriophages can be regarded as a further confirmation for the presence of specific bacterial genera. In our experience, detection of DNA phages and/or an increased number of unmapped reads above 2% indicate elevated bacterial titers and thus a significant bacterial infection of clinical relevance. In one of our previous papers, we analyzed FFPE lung tissues by the methodology described here (Nienhold et al., 2020). This shows that the method is in principle suitable for detection of DNA viruses in FFPE material. The amount of reads from bacteriophages followed a similar linearity as the reads of the respective bacterial genus in the same series of samples. Indeed, bacteriophages were detected in some FFPE lung tissue samples with high titer bacterial infections. In our sensitivity/linearity test, bacteriophages were detected in samples containing more than 1000 genomes of the respective bacterial genus. These results show that, with our procedure, we are able to lyse bacterial cells within FFPE samples, and results generated by the dilution series of mock communities are reproducible in human FFPE samples.

Overall, we recommend parallel microscopic examination of tissues to interpret metagenomics results in context of local histomorphology. This will create a deeper understanding of how specific bacterial species may evoke specific histological patterns and clinical symptoms. The challenges in molecular infectious pathology require a new type of pathologist. In the future, specialized pathologists need to combine morphological findings with knowledge in molecular and cell biology, genetics, biochemistry and bioinformatics (Mullauer, 2017).

Debesa-Tur *et al.* published a first study on WGS-based metagenomics on FFPE samples (Debesa-Tur et al., 2021). While our methodology is similar to the method described in that paper, our study is still the first study that focuses specifically on detection of bacterial pathogens in FFPE samples, rather than the microbiome. This is an important aspect, particularly for pathologists. We also apply this method to lung tissues with lower microbial burden, compared to colon that was analyzed by Debesa-Tur *et al.* This is an important extension of the method, because WGS metagenomics assays have mostly been used for samples with a comparatively high amount of bacteria and/or low amount of host cells, such as liquor or synovial fluids (Thoendel et al., 2018; Blauwkamp et al., 2019; Piantadosi et al., 2021). In line with this notion, it was assumed that shotgun sequencing may not be a viable replacement for 16S rRNA sequencing when characterizing blood or biopsy microbiomes, where there is more host DNA contamination and relatively low bacterial biomass (Hillmann et al., 2018). However, our study now demonstrates that WGS-based metagenomics is usable for these tissues.

The metagenomics workflow for identification of pathogens from FFPE human tissues as described here has several limitations. Due to the nature of routine tissue handling and work-up, there is always a risk of accidental contamination of samples. This risk is mitigated by the lower sensitivity of the metagenomics analysis compared to species-specific qPCRs. A further limitation is that RNA viruses escape detection as this metagenomics assay is based on the analysis of DNA. In the future, it might be possible to extend our workflow by an additional RNA extraction and reverse transcription step. However, it can be expected that RNA yields from small biopsies will be low, and substantial effort will be required to optimize the sensitivity range.

A further limitation is that we validated this assay on human lung tissue only. It is expected that the metagenomics analysis works best in tissues that are sterile or have only a restricted microbiome (Baughman et al., 1987; Charlson et al., 2011). In tissues with a complex microbial background the distinction of relevant pathogens from commensals requires a defined threshold for subtraction of background species that do not contribute to relevant infections. For instance, in skin (Grice et al., 2009; Chang et al., 2018) and intestinal (Qin et al., 2010) tissues, metagenomics analysis will be limited to the detection of one or more prominent species that are clearly above the background signal of commensals. However, the definition of the background threshold will be arbitrary as it depends on the tissue analyzed and the bacterial species detected. Further, relevant species that might give rise to subclinical low-titer infections such as *Mycobacterium tuberculosis* must be exempted from this subtraction (Caulfield and Wengenack, 2016). Therefore, the metagenomics method is inappropriate or limited for detection of these infections. This is a caveat specific for our method described here and not a limitation of metagenomics analyses in general. Known commensals can be subtracted from a complex, abundant microbiome based on species information, and the remaining reads can be analyzed for potential pathogenic species irrespective of their abundance. Furthermore, it is possible to enhance the sensitivity of metagenomic methods through pre-enrichment methods. Amplicon-based 16S rRNA gene sequencing methods might be more sensitive and better suitable to detect low-titer infections, however, introduce a detection bias because targeted amplification limits the number of species that can be detected (Johnson et al., 2019).

Finally, the selection of a specific reference database directly impacts the range of detectable pathogens, as the number of covered species varies between databases (DeSantis et al., 2006; Quast et al., 2013; Sayers et al., 2022). The software used in this study, the CLC genomics workbench, is customizable as it allows to import any database and raw data of choice, including raw data from all common NGS technology providers (e.g. Ion Torrent, Illumina, Pacific Biosciences, Oxford Nanopore Technologies) to generate a custom reference database. This analysis can be performed locally, i.e. it can be used in settings where legal restrictions do not allow uploading and analyzing patient data on shared commercial server space.

In summary, the metagenomics analysis workflow presented here is to the best of our knowledge the first method that enables rapid and unbiased identification of pathogens in FFPE tissue samples. This method is ideal for cases where an infection is suspected based on clinical or histological results, yet species-specific PCRs of common pathogens are negative. This metagenomics assay enables the identification of unknown bacterial pathogens and may generate novel hypotheses about the conditions under which commensal bacteria may become pathogenic. In combination with histomorphological results, this method is expected to provide deeper insights into the mechanisms of bacterial infections in tissues.

## Data availability statement

The datasets presented in this study can be found in online repositories. The names of the repository/repositories and accession number(s) can be found below: https://www.ebi.ac.uk/ena, PRJEB53459

## Ethics statement

The studies involving human participants were reviewed and approved by Ethics Committee of Northwestern and Central Switzerland (Project-ID 2020-00629). Written informed consent for participation was not required for this study in accordance with the national legislation and the institutional requirements.

## Author contributions

RN, TJ and KM jointly conceived the study, performed data interpretation, and prepared the manuscript. NM, AF, AG and JK performed research and collected data. NS and CH collected autopsy specimens and patient data. VZ, NW and GC performed the histomorphological evaluation. SG collected and processed clinical isolates. BH generated additional data for the revised version of the manuscript. All authors contributed to the article and approved the submitted version.

## Funding

This study was funded by the Botnar Research Centre for Child Health (BRCCH), FTC-2020-10.

## Conflict of interest

Author Tobias Junt is employed by Novartis Institutes for BioMedical Research (NIBR), Basel, Switzerland.

The remaining authors declare that the research was conducted in the absence of any commercial or financial relationships that could be construed as a potential conflict of interest.

## Publisher’s note

All claims expressed in this article are solely those of the authors and do not necessarily represent those of their affiliated organizations, or those of the publisher, the editors and the reviewers. Any product that may be evaluated in this article, or claim that may be made by its manufacturer, is not guaranteed or endorsed by the publisher.

## Supplementary material

Supplementary Figure 1
*In silico* down-sampling of metagenomics reads on six FFPE samples with bacterial infections. *Left*, bar graphs, metagenomics analysis results from the original NGS analysis (1.0x10^7^ to 4.4x10^7^ total reads per sample). *Right*, bar graphs, metagenomics analysis results from 100 randomly picked sets of 5x10^6^ reads from the original NGS data of each sample.

Supplementary Figure 2 Effect of the metagenomics analysis pipeline on raw reads from mock communities. **(A)** Number of reads at individual workflow stages. **(B)** Proportion of reads from previous workflow stage passing the indicated step in the analysis pipeline. **(C)** Number of unmapped reads in various dilutions and original concentration (undil.) of all four mock communities. Plots of **(A–C)**: Box shows range from first to third quartile and median, whiskers display minimum and maximum. **(D)** Sensitivity of qPCR and metagenomics analysis in detecting known bacterial pathogens and reference species at different concentrations of mock communities.

Supplementary Figure 3 Effect of the metagenomics analysis pipeline on raw reads from human FFPE lung tissue samples. **(A)** Number of reads at individual workflow stages. **(B)** Proportion of reads from previous workflow stage passing the indicated step in the analysis pipeline. Plots of **(A, B)**: Box shows range from first to third quartile and median, whiskers display minimum and maximum. **(C)** Genetic origin of sequence information interpreted from read mapping to the human genome GRCh37 (hg19) or taxonomic profiling of non-human reads. Reads, which could not be assigned to human, bacteria or viruses, are labeled as unidentified. Positive, samples positive for bacterial species; Negative, samples without bacterial species, as determined by metagenomics analysis. Bar graph displays median and standard deviation for all 73 (29 positive and 44 negative) samples. Grey bar=median. **(D)** Neutrophilic infiltration in samples positive for *Staphylococcus aureus*, as determined by qPCR, in dependence of the detection of S. aureus by metagenomics analysis.

Supplementary Figure 4 Sunburst graph depicting phylogeny and quantity of bacteria detected in the most complex case of infection in a human FFPE lung. Each circle represents a phylogenic level, starting with the kingdom level in the inner ring and ending with the species level at the outer rim.
