# Supplementary material for: Unbiased screen for pathogens in human paraffin-embedded tissue samples by whole genome sequencing and metagenomics
Source: Front Cell Infect Microbiol. 2022 Sep 20;12:968135. doi: 10.3389/fcimb.2022.968135 (PMC9530700; doi:10.3389/fcimb.2022.968135)

Supplementary Figure 1

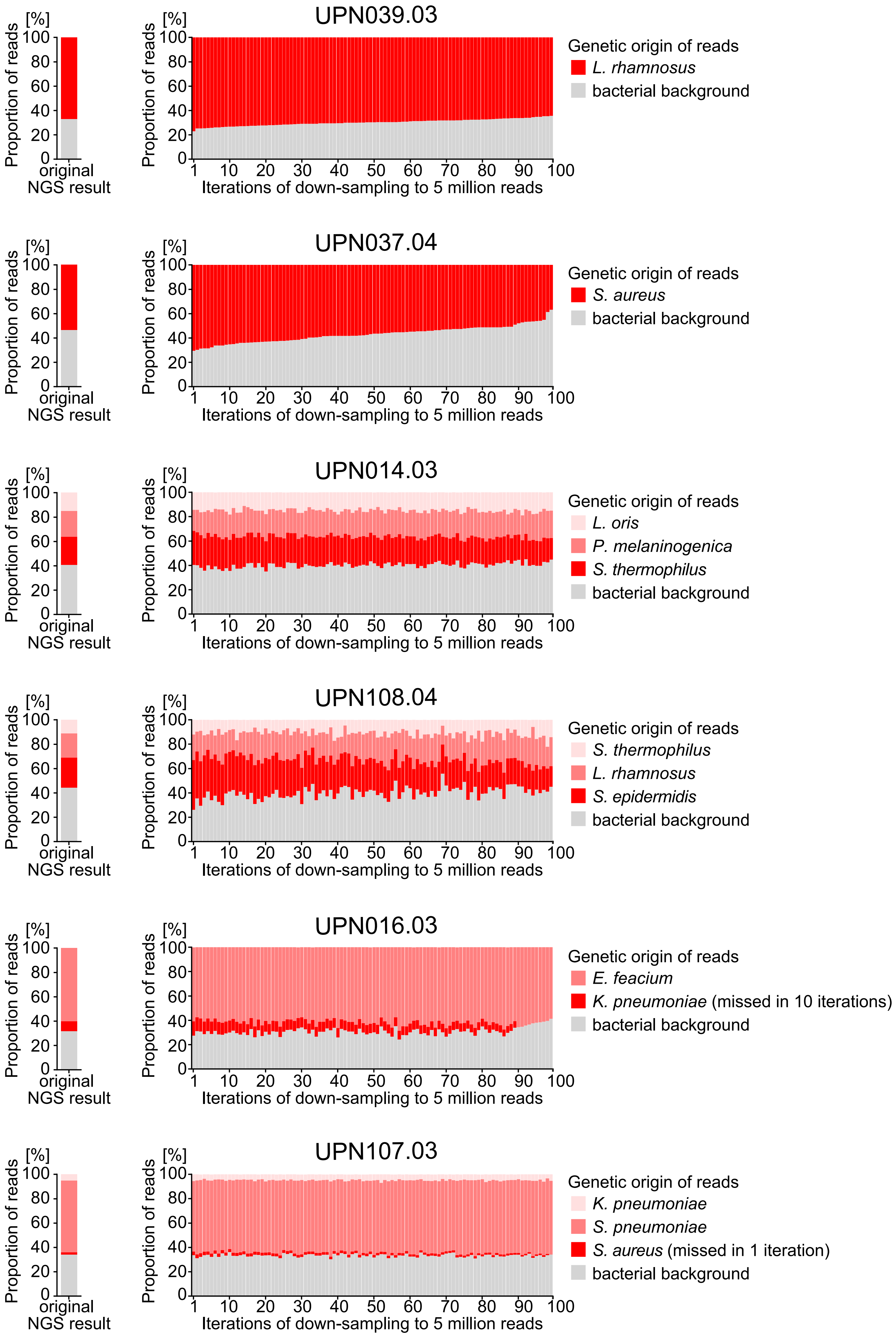

Supplementary Figure 2

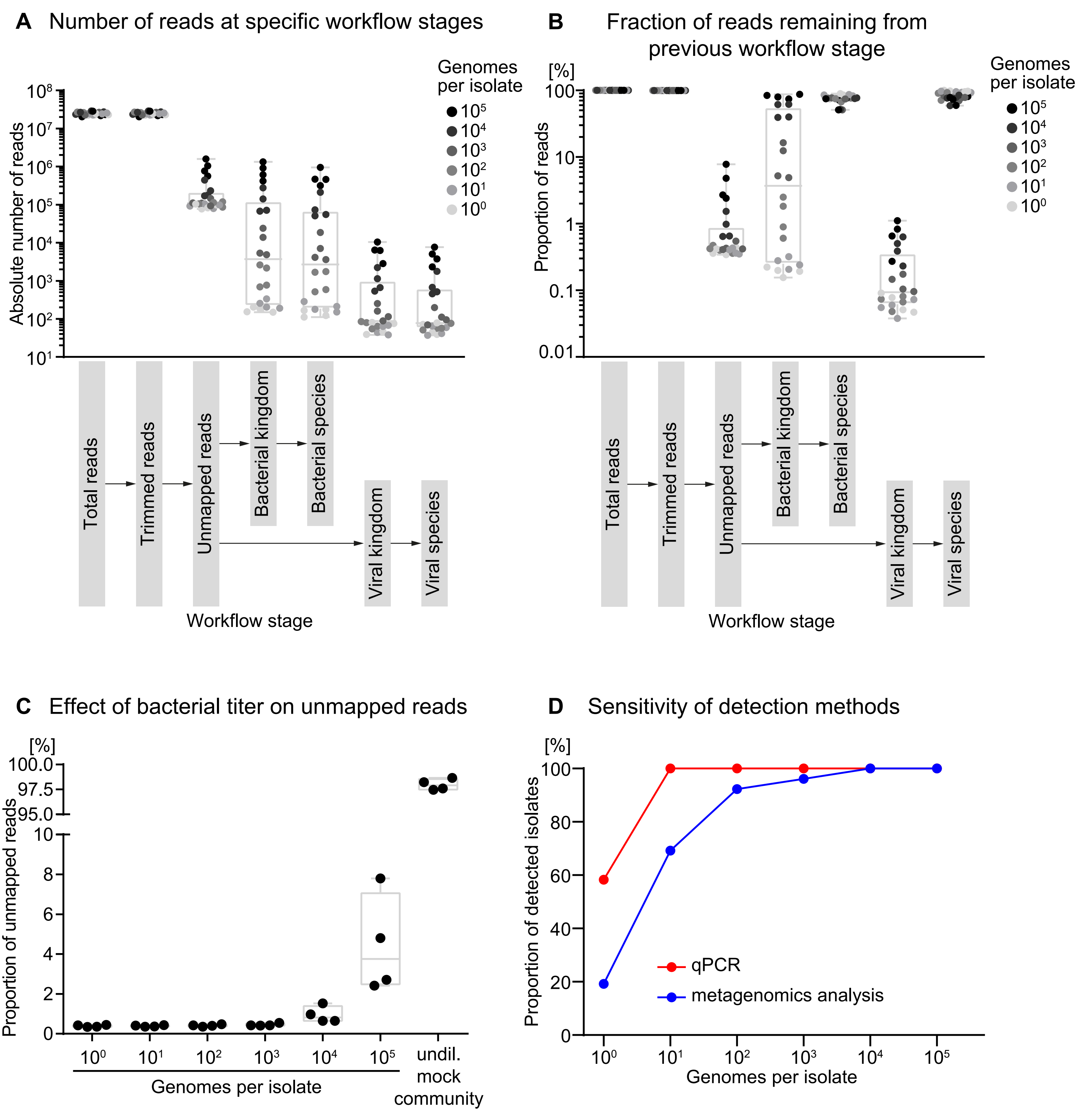

Supplementary Figure 3

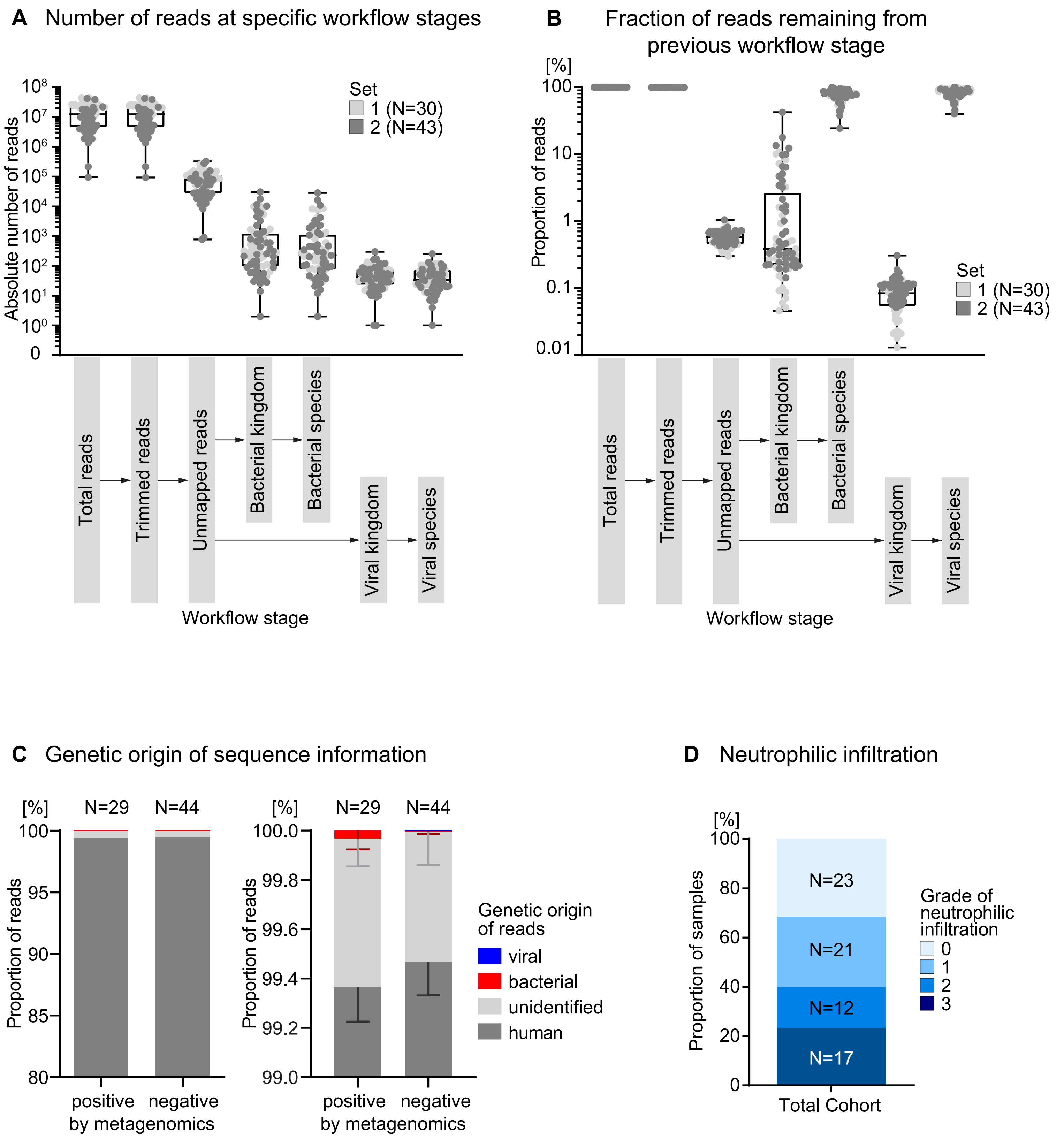

Supplementary Figure 4

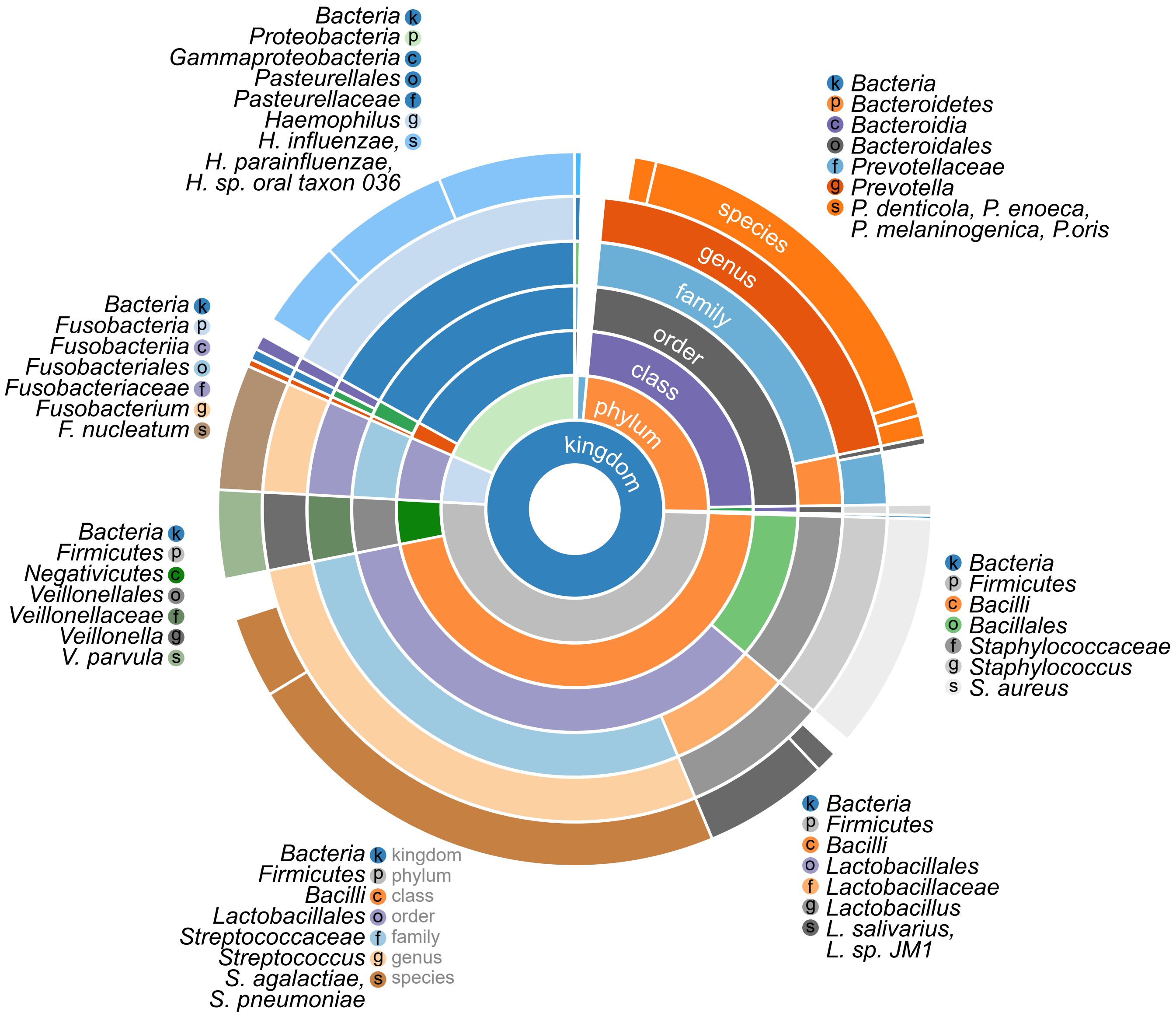

Supplement: Supplementary Figure 1 — In silico down-sampling of metagenomics reads on six FFPE samples with bacterial infections. Left, bar graphs, metagenomics analysis results from the original NGS analysis (1.0x10^7^ to 4.4x10^7^ total reads per sample). Right, bar graphs, metagenomics analysis results from 100 randomly picked sets of 5x10^6^ reads from the original NGS data of each sample. [file DataSheet_1.pdf]
